# Cryoablation for Barrettʼs esophagus with dysplasia and eosinophilic esophagitis: A novel approach for a rare dual condition

**DOI:** 10.1055/a-2598-3788

**Published:** 2025-06-13

**Authors:** Dario Quintini, Caterina Stornello, Monica Franco, Giulia Peserico, Sabina Grillo, Mariagrazia De Palo, Alberto Fantin

**Affiliations:** 1 Gastroenterology Unit, Veneto Institute of Oncology IOV-IRCCS, Padua, Italy

We present the case of a 58-year-old man affected by long-segment Barrettʼs esophagus (BE-C10M10, according to the Prague classification) and eosinophilic esophagitis (EoE) ( Video 1 ). He had been followed for several years since the diagnosis of BE in 2007, until 2023, when random biopsies revealed focal low grade dysplasia and high grade dysplasia. The diagnosis of EoE was occasionally made in 2016, but the patient was asymptomatic, and he was never treated for that.

A novel cyroablation procedure for Barrett’s esophagus with dysplasia and eosinophilic esophagitis.Video 1

At that point, BE ablation was indicated, but radiofrequency ablation (RFA) is relatively contraindicated in patients with EoE due to the lack of studies testing the safety of this procedure in this cohort of patients, and because both RFA and EoE are risk factors for esophageal strictures. To our knowledge, no association between EoE and BE requiring treatment has been previously reported in the literature.

After a multidisciplinary board discussion, cryoablation (C2 CryoBalloon Ablation; PENTAX Medical, Tokyo, Japan) for BE was proposed after a short course of budesonide to prevent EoE-related complications. The patient was admitted and treated with high-dose proton pump inhibitors (40 mg twice daily) and analgesics on demand. During the procedure, argon plasma coagulation was used to mark the gastroesophageal junction, and cryoablation was performed circumferentially on the lowest 4 cm of BE (16 total applications). No complications occurred and oral feeding started on day 1 with a soft diet, which continued for two weeks. The patient reported only mild dysphagia during the first five days. He was discharged on day 5 after the procedure. For home treatment, 30 mg lansoprazole three times daily for one week, then 30 mg twice daily for one month, and 30 mg once daily thereafter, in association with 2 g sucralfate gel twice daily, were prescribed. After three months, a new session was performed, treating the uppermost 3 cm of BE; the same patient management was utilized. No evidence of strictures was found and no post-procedural adverse events occurred. A new session was planned after three months.

Endoscopy_UCTN_Code_TTT_1AO_2AN

